# Acoustic Analyses of Speech Sounds and Rhythms in Japanese- and English-Learning Infants

**DOI:** 10.3389/fpsyg.2013.00057

**Published:** 2013-02-28

**Authors:** Yuko Yamashita, Yoshitaka Nakajima, Kazuo Ueda, Yohko Shimada, David Hirsh, Takeharu Seno, Benjamin Alexander Smith

**Affiliations:** ^1^Graduate School of Design, Kyushu UniversityFukuoka, Japan; ^2^Department of Human Science, Center for Applied Perceptual Research, Kyushu UniversityFukuoka, Japan; ^3^Graduate School of Asian and African Studies, Kyoto UniversityKyoto, Japan; ^4^Faculty of Education and Social Work, University of SydneySydney, NSW, Australia; ^5^Faculty of Design, Institute for Advanced Study, Kyushu UniversityFukuoka, Japan; ^6^Department of Design, Architecture and Planning, University of SydneySydney, NSW, Australia

**Keywords:** infant vocalization, speech development, spectral fluctuations, factor analysis, speech rhythm

## Abstract

The purpose of this study was to explore developmental changes, in terms of spectral fluctuations and temporal periodicity with Japanese- and English-learning infants. Three age groups (15, 20, and 24 months) were selected, because infants diversify phonetic inventories with age. Natural speech of the infants was recorded. We utilized a critical-band-filter bank, which simulated the frequency resolution in adults’ auditory periphery. First, the correlations between the power fluctuations of the critical-band outputs represented by factor analysis were observed in order to see how the critical bands should be connected to each other, if a listener is to differentiate sounds in infants’ speech. In the following analysis, we analyzed the temporal fluctuations of factor scores by calculating autocorrelations. The present analysis identified three factors as had been observed in adult speech at 24 months of age in both linguistic environments. These three factors were shifted to a higher frequency range corresponding to the smaller vocal tract size of the infants. The results suggest that the vocal tract structures of the infants had developed to become adult-like configuration by 24 months of age in both language environments. The amount of utterances with periodic nature of shorter time increased with age in both environments. This trend was clearer in the Japanese environment.

## Introduction

During the first 2 years of age, the human speech production mechanism develops rapidly. Various anatomic structures of the vocal tract grow to 55–80% of adult size by 18 months of age (Vorperian et al., 2005). Corresponding to the growth of the vocal tract as well as the control of places and manners of articulation, infant vocalization changes from cooing (vowel-like sounds) to babbling (e.g., da-da or ma-ma), and then to words similar to adult speech during the first 2 years of life.

A number of studies have explored the acoustic characteristics in infant speech spectra, such as formants or spectral peaks of vowels (e.g., Buhr, 1980; Lieberman, 1980; Bond et al., 1982; Kent and Murray, 1982; Gilbert et al., 1997; Rvachew et al., 2006; Ishizuka et al., 2007); these acoustic characteristics reflect the development of the vocal tract and the acquisition of places and manners of articulation. For example, Gilbert et al. (1997) explored developmental characteristics of formant 1 (F1) and formant 2 (F2) produced by four young English-learning children between 15 and 36 months of age. The results revealed that F1 and F2 were relatively stable during the period of 15–21 months and their frequencies decreased significantly between 24 and 36 months. Gilbert et al. (1997) suggested that the vocal tract length and pharyngeal space increased whereas nasal cavity influence decreased, which would probably result in relatively stable F1 and F2 during the period of 15–21 months. Bond et al. (1982) analyzed F1 and F2 of English front and back vowels between 17 and 29 months, and showed that vowel formants shifted in accordance with vowel space expansion with age. Ishizuka et al. (2007) also explored longitudinal developmental changes (4–60 months of age) in spectral peaks of vowels with two Japanese-learning infants. The results showed that a categorically separated vowel space is formed by around 20 months of age, and that the speed of vowel space expansions is rapid by around 24 months of age. These studies supported the view that there are rapid developmental changes in acoustic characteristics during the first 2 years of age corresponding to anatomical development of the vocal tract and manners and places of articulation.

In addition to the acoustic characteristics in infant speech, increasing attention has been devoted to temporal periodicity (e.g., Oller, 1986; Davis and MacNeilage, 1995; Davis et al., 2000; Kouno, 2001; Nathani et al., 2003; Petitto et al., 2004; Dolata et al., 2008). This interest has been caused by the statement that consonant-vowel (CV) sequences in babblings are simply determined by open-close mandibular oscillation, which gives listeners the perceptual impression of temporal regularity (e.g., Davis and MacNeilage, 1995; Oller, 2000). Dolata et al. (2008) explored the repetition of CV forms in reduplicative vocal babblings obtained from English-learning infants (7–16 months of age) and reduplicated syllables from adult speakers. The results showed that the mean syllable duration in vocal babblings was 329.5 ms and 95% of total durations were between 250 and 425 ms. For adult speakers, the mean syllable duration was 189 ms, which was shorter than that of infant utterances. Nathani et al. (2003) investigated normally hearing and deaf infants at prelinguistic vocal development. For normally hearing infants, the mean nonfinal syllable durations decreased from 378 to 316 ms, and final syllable durations decreased from 527 to 355 ms. Final syllable length ratios for normally hearing infants decreased across age whereas it was relatively stable for deaf infants. The results suggested that the rhythmic organization was influenced by the auditory status and the level of vocal development. Kouno (2001) reported that syllable duration of two- or three-syllable words gradually decreased to be less than 420 ms in babbling forms and less than 330 ms in word forms in Japanese-learning infants by around 20 months of age. Both studies (Kouno, 2001; Nathani et al., 2003) showed gradual development in that the syllable duration in infant vocalizations became shorter across age.

Some studies attempted to find language-related aspects of temporal periodicity in early word production period. A representative series of Vihman (1991, Vihman et al. 1998, 2006), Vihman and de Boysson-Bardies (1994) explored speech rhythm in infant production from different language backgrounds. For example, Hallé et al. (1991) investigated duration patterns in disyllabic vocalization in either word or babbling forms with Japanese- and French-learning infants by around 18 months of age. Final syllable lengthening, which reflected duration characteristics in French, was found in French-learning infants, whereas it was absent for Japanese-learning infants: Language-related aspects of prosodic patterns were already found in infant utterances in these linguistic environments. Vihman et al. (1998) examined disyllables obtained from English- and French-learning infants in the late single-word period (13–20 months of age). The tendency that the second vowel duration was longer than the first vowel duration was adult-like in French-learning infants, whereas each syllable was at considerably higher level of variability, which less closely matched to prosodic patterns in adult speech, in English-learning infants. There was also individual variability for English-learning infants. Vihman et al. (1998) considered children’s differing learning strategies, and argued that each child filtered the input of language, and attempted to reproduce words based on their favored word production templates. Language-related aspects were found while there was variability of syllable duration in the early word production period.

Although these studies shed light on the developmental changes in acoustic characteristics and temporal periodicity, they had the following problems: (1) Formant frequency analysis (e.g., Buhr, 1980; Lieberman, 1980; Bond et al., 1982; Kent and Murray, 1982; Gilbert et al., 1997; Ishizuka et al., 2007), which was most frequently used, is employed basically to detect only vowel sounds in order to obtain knowledge for linguistic development. There has been a lack of acoustic analysis which measures the whole pattern of spectral fluctuations. (2) Speech samples to observe temporal periodicity were limited to disyllabic vocalizations (e.g., Hallé et al., 1991; Vihman et al., 1998; Davis et al., 2000). There was no automatic measurement to identify temporal periodicity, and thus phoneticians judged duration by looking at speech waveforms, which might have be subjective.

**Figure A1:**
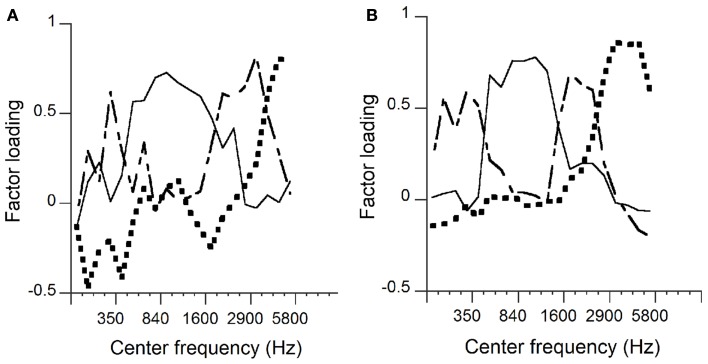
**The graph in (A) shows the results of factor analysis from adult Japanese speakers (*N* = 10), and the graph in (B) shows the results from adult English speakers (*N* = 10)**. The total number of sentences was 20 for each language group. The solid lines, dashed lines, and dotted lines represent factors 1, 2, and 3, respectively.

In the present study, a critical-band-filter bank was used to analyze the spectral fluctuations and temporal periodicity in infants’ utterances. A practical way to analyze speech signals is to separate them into a certain number of narrow frequency bands as in a historical (traditional) vocoder system, and to observe the temporal power fluctuation in each frequency band. The notion of critical bands, which reflects basic characteristics of the auditory system (see, e.g., Fletcher, 1940; Zwicker and Terhardt, 1980; Patterson and Moore, 1986; Unoki et al., 2006; Fastl and Zwicker, 2007; Moore, 2012), seemed convenient for our present purpose, because the power fluctuations in 15–22 critical bands contain enough information to make speech almost fully intelligible. Ueda and Nakajima (2008) performed factor analyses of the spectral fluctuations in speech sounds of different languages, utilizing a critical-band-filter bank. The same three factors appeared in Japanese and English, which were replicated for a far smaller number of speech samples (see Figure A1 in Appendix). The critical-band-filter bank analysis seemed applicable to Japanese- and English-learning infant speech in order to detect the whole pattern of spectral fluctuations. We were particularly interested in what age of life the factors as in adults’ speech would appear in infant speech.

**Figure A2:**
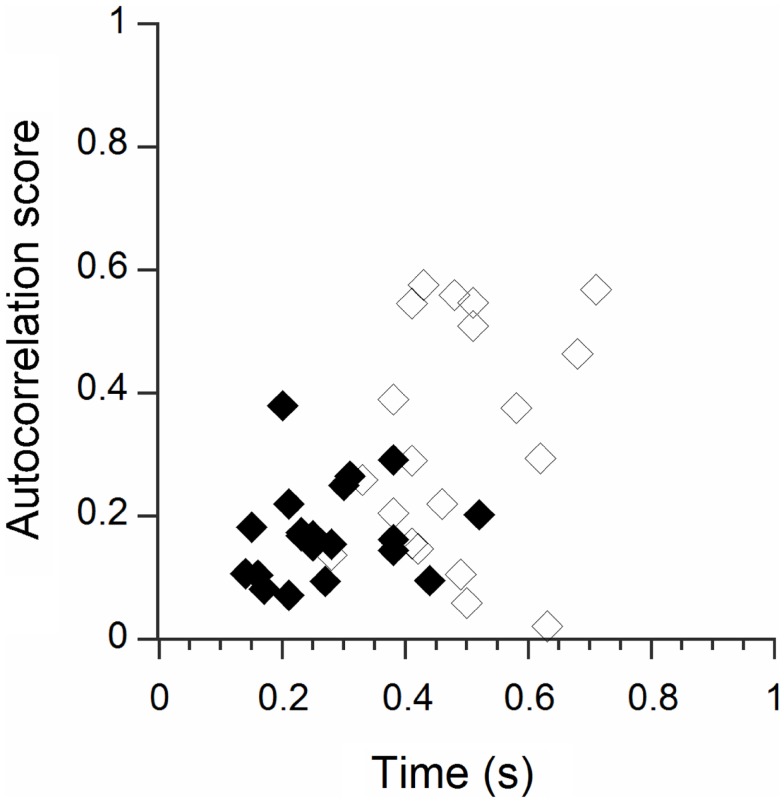
**The graph shows the autocorrelation score and time of the first peak for each sample of adult Japanese speakers (black square) and adult English speakers (white square)**. The acoustic method clarified difference in temporal periodicity between Japanese and English.

As a next step, we explored the temporal periodicity in infant speech obtained from Japanese- and English-learning infants. The speech samples in the current study were not limited to disyllabic vocalization. We used all the speech samples (≥1.5 s) in order to explore the whole pattern of developmental changes. We utilized the temporal periodicity of the factor scores that summarizes power fluctuations of speech sounds in the outputs of critical-band filters, instead of measuring temporal intervals in speech waveforms by the eye. Japanese and English adult speech samples in a database were first analyzed, and the validity of this method was proved (see Figure A2 in Appendix). Thus, we applied this method to identify the temporal periodicity in infant speech.

Three ages, 15, 20, and 24 months, were selected for the following reasons. The various vocal tract structures, predominantly pharyngeal/posterior structure, achieve 55–80% of the adult size by 18 months of age (Vorperian et al., 2005). In addition to the development of vocal tract, lexical development is in rapid progress from 12 to 18 months of age. Many infants over this period become capable of producing at least 50 meaningful words, which is so called “50-word stage” (MacNeilage et al., 2000). After “50-word stage,” there is an explosion of phonetic diversification due to the better control of manners and places of articulations to produce a variety of consonant sounds, and expansion of the vowel spaces to include diverse vowel types (Kern et al., 2010). Thus, around the age of 15 months, the vocal tract is in the process of rapid development and this corresponds to a period of rapid lexical development (12–18 months), while infants from 20 to 24 months of age become capable of diversifying phonetic inventories and form some sentences to convey more complex messages. Thus, the period of 15–24 months of age seemed appropriate to explore significant changes in infant speech development.

The questions of infant speech development were addressed as follows:

(1) How do spectral fluctuation and temporal periodicity in infant speech change between 15 and 24 months of age?
(2) Are the developmental changes of speech in the acoustic domain similar in Japanese- and English-learning infants?

## Materials and Methods

### Infant participants

Participants included five typically developing infants at 15 months of age (three girls and two boys), five infants at 20 months of age (three girls and two boys), and five infants at 24 months of age (three girls and two boys) from Japanese-speaking families. Five typically developing infants at 15 months of age (three girls and two boys), five infants at 20 months of age (two girls and three boys), and four infants at 24 months of age (three girls and one boy) were from English-speaking families. The Japanese-learning infants were being raised by monolingual Japanese adult speakers. The English-learning infants were being raised by monolingual English adult speakers or adult speakers whose first language is English. For all Japanese-learning infants, their weight was over 8, 10, and 9 kg and height was over 76, 83, and 82 cm at 15, 20, and 24 months of age, respectively. For all English-learning infants, their weight was over 10, 11, and 10 kg and their height was over 78, 84, and 84 cm at 15, 20, and 24 months of age, respectively. This showed that all infants exhibited normal physical development. Parental consent forms and information sheets were provided to a parent of each infant. The procedures required for the project and the time involved were explained. Parental consent forms from each parent were received.

### Recordings

Utterances were recorded in a quiet room in each infant’s home for about 2 h a month. Special care was taken to keep each infant in a normal environment at home. A digital sound recorder (Roland, R-09HR or TEAC, DR-07) was set to 44.1-kHz sampling and 16-bit linear quantization. The recorder was placed on a pillow in order to prevent vibration and reverberation. It was kept at least 1 m away from the infant in order to stabilize the recording level. The parent or parents were instructed to behave in a usual manner and to do daily activities during the recording process. No specific procedures to elicit infant vocalization were utilized.

### Speech samples

One of the authors and two students in the Department of Acoustic Design and Human Science course at Kyushu University extracted utterances from each 2-h recording, using audio software (Syntrillium, Cool Edit 2000, or Adobe, Audition) based on the following criteria:

1. Silent parts of 75 ms before and after each utterance were included.
2. If a silent part between two potential utterances was shorter than 1200 ms, the whole pattern was considered a single utterance. Since we were particularly interested in rhythmic patterns in speech, we calculated autocorrelations of factor scores up to 1 s. This prohibited us from discarding silent intervals shorter than 1 s. For assurance, we included all silent intervals shorter than 1200 ms as part of the utterances to be analyzed.
3. If a single utterance was separated by adult speech or background noise, the separated parts were analyzed as different utterances.
4. If an utterance was overlapped by adult speech or background noise from toys or other objects, it was excluded from analysis.
5. Anomalous vocal signals, such as laughter, crying, squeals, growls, and shrieking were excluded.

We constructed a database consisting of utterances of Japanese- and English-learning infants. Speech samples longer than 1.5 s in this database represented 25, 30, and 54% of all utterances for Japanese-learning infants at 15, 20, and 24 months of age, respectively, and 23, 27, and 59% of all utterances for English-learning infants at 15, 20, and 24 months, respectively.

Table 1 presents information regarding the number of utterances and the average duration of utterances obtained for each infant. In total, 484, 474, and 586 utterances were collected from Japanese-learning infants at 15, 20, and 24 months, respectively; 529, 465, and 426 utterances were collected from English-learning infants at 15, 20, and 24 months, respectively.

**Table 1:** **Number and average duration of utterances**.

	Months of age	Number of utterances	Average duration of utterances (s)	Standard deviation (SD)
**JAPANESE-LEARNING INFANTS**				
JF2	15	98	1.98	1.67
JF6	15	132	1.08	1.03
JM3	15	111	0.99	0.64
JM4	15	69	1.18	0.78
JM7	15	74	1.67	1.36
Overall		484	1.38	1.07
JM1	20	90	1.15	0.96
JF2	20	102	1.22	0.86
JF3	20	95	1.16	1.14
JM3	20	85	1.79	1.26
JF1	20	102	1.30	1.10
Overall		474	1.32	1.06
JF2	24	101	1.83	0.9
JF3	24	130	1.49	0.74
JF6	24	124	1.39	0.67
JM1	24	123	1.52	0.8
JM3	24	108	1.45	0.65
Overall		586	1.53	0.75
**ENGLISH-LEARNING INFANTS**				
EF1	15	99	1.71	1.98
EF3	15	105	1.16	1.03
EM3	15	114	1.09	0.93
EF2	15	120	0.98	0.77
EM1	15	91	0.75	0.52
Overall		529	1.19	1.05
EF1	20	107	1.17	0.62
EF2	20	78	1.26	0.93
EM2	20	73	1.18	0.84
EM4	20	121	0.9	0.69
EM1	20	86	0.99	0.67
Overall		465	1.10	0.73
EF1	24	96	1.71	0.79
EF2	24	85	2.30	0.91
EF07	24	107	1.41	0.74
EM06	24	138	1.73	0.82
Overall		426	1.79	0.81

### Speech analysis

All the speech signals were analyzed using the same approach as in Ueda and Nakajima (2008). A bank of critical-band filters was constructed. The total passband of the filter bank ranged from 100 to 12,000 Hz, and the center frequencies of the filters ranged from 150 to 10,500 Hz. The cutoff frequencies of the critical-band filters were based on Zwicker and Terhardt (1980). Each filter was constructed as concatenate convolutions of an upward frequency glide and its temporal reversal. Both sides of the filters had slopes steeper than 90 dB/oct. Each filter output was squared, smoothed with a Gaussian window of σ = 20 ms, and sampled at every 1 ms. Factor analyses were performed based on the correlation matrices between the power fluctuations of the 22 critical-band filters. In each age/language group, the average levels of all the speech samples were adjusted to be equal to each other, and the adjusted samples were connected in time for factor analysis. The total duration of the connected signals was 667, 626, and 897 s for the Japanese-learning infants and 630, 512, and 763 s for the English-learning infants, at 15, 20, and 24 months of age, respectively. Correlation-based (normalized) analysis was performed; varimax rotation followed principal component analysis. The number of factors was set at two or three in order to compare the present results with Ueda and Nakajima’s (2008) results.

In the following analysis, the autocorrelation functions were obtained in order to observe temporal periodicity in the factor scores. The correlation between the *n*th and the (*n* + *k*)th sample in a time series of *N* samples was calculated as follows:

$$\begin{array}{llll} r\left(k\right) & =\frac{\sum_{n=1}^{N-k}\left(x_{n}-{\overset{/}{x}}_{1}\right)\left(x_{n+k}-{\overset{/}{x}}_{k+1}\right)}{\sqrt{{\sum_{n=1}^{N-k}\left(x_{n}-{\overset{/}{x}}_{1}\right)}^{2}}\cdot \sqrt{{\sum_{n=\kappa +1}^{N}\left(x_{n}-{\overset{/}{x}}_{k+1}\right)}^{2}}}, & & \\ \text{where}\;{\overset{/}{x}}_{1} & =\frac{\sum_{n=1}^{N-k}x_{n}}{N-k},\;\text{and} & & \\ {\overset{/}{x}}_{k+1} & =\frac{\sum_{n=k+1}^{N}x_{n}}{N-k}. & & \end{array}$$

The autocorrelation function of the temporal distance τ was defined as

$$R\left(\tau \right)=r\left(\tau \cdot f_{\text{s}}\right),$$

where *f*_s_ represents the sampling frequency; *R*(τ) was defined only when τ ·*f*_s_ was an integer.

In the factor analysis, factor scores were sampled at every 1 ms. We used speech samples ≥1.5 s and observed temporal periodicity in factor scores by calculating autocorrelations up to 1 s. There was always a factor including a frequency range of 1000–1600 Hz, and this factor seemed to be related to vowel-like sounds (Nakajima et al., 2012); the autocorrelation of this factor (factor scores as a function of time) was calculated for each utterance in order to observe a global pattern of temporal periodicity, if any. The amplitude of the first peak above zero was taken as the representative of an autocorrelation score. If there was no peak above zero, the autocorrelation function was considered to be without a peak.

## Results

### Factor analyses

**Figure 1:**
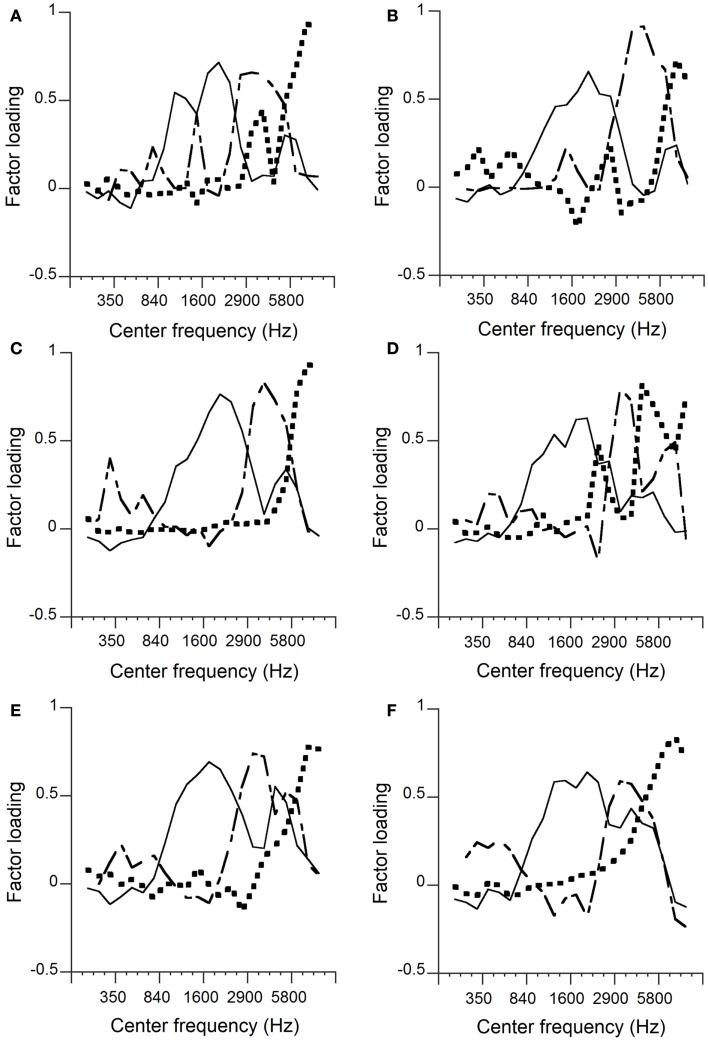
**Factor analyses**. Japanese-learning infants at 15 months of age **(A)**, English-learning infants at 15 months **(B)**, Japanese-learning infants at 20 months **(C)**, English-learning infants at 20 months **(D)**, Japanese-learning infants at 24 months **(E)**, and English-learning infants at 24 months **(F)**. The solid lines, dashed lines, and dotted lines represent factors 1, 2, and 3, respectively.

Figures 1A–F show the results obtained from Japanese- and English-learning infants at 15, 20, and 24 months of age. Factor 1 related to a frequency range around 1600 Hz, factor 2 was bimodal surrounding factor 1, and was related to frequency ranges around 350 Hz and around 4000 Hz, and factor 3 was related to high frequency ranges.

**Figure A3:**
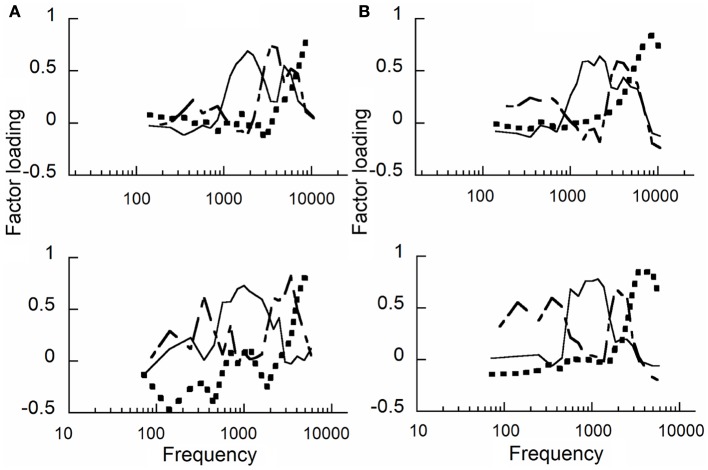
**The graphs in (A) show the results from Japanese-learning infants at 24 months (upper) and adult Japanese speakers (lower), and the graphs in (B) show the results from English-learning infants at 24 months (upper) and adult English speakers (lower)**. The solid lines, dashed lines, and dotted lines represent factors 1, 2, and 3, respectively. Adult speakers’ data are from Figure A1. The use of logarithmic frequency scales is helpful to compare the configurations of the factors in infant and adult speech. The horizontal axis in the graph of adult speech was shifted by 1.7 times. If a point in an upper graph and another point in a lower graph agreed with each other on the horizontal location, the frequency in the upper graph is 1.7 times as high as that in the lower graph. The graphs showed that the configurations of the three factors in infant speech were in correspondence with those in adult speech. Roughly speaking, the frequency boundaries for the infant data were higher by a factor around 1.7 times. This tolerably corresponds to the fact that infants’ articulation organs at this age are 55–80% in size compared with the adults’ articulation organs (Vorperian et al., 2005).

The factor loadings of factors 1–7 or 1–8 whose original principal components always exhibited eigenvalues greater than 1, were observed. The cumulative contributions obtained from the data for each language/age group were 50–57% for the seven or eight components. For comparison with adult speech, two or three factors were chosen. The Cumulative contributions were 30–32% for the first three components. The first three components showed clear correspondence with the adults’ results for Japanese-learning infants at 20 and 24 months, and English-learning infants at 24 months. The second or third factor did not show clear correspondence with any particular frequency ranges for Japanese-learning infants at 15 months or with English-learning infants at 15 and 20 months. For older infants, factor 1 was surrounded by factor 2, which was bimodal, and factor 3 was specifically related to the highest frequency range. If factor loadings are indicated against frequency represented logarithmically, the configurations of the three factors in the infant speech at 24 months of age are well in correspondence with those in the adult speech in both linguistic environments (see Figure A3 in Appendix).

Peaks of the curves represented relatively high factor loadings, and we considered the crossover frequency of two adjacent curves as an indication of the boundary between the corresponding factors. Table 2 shows the obtained boundaries as represented by the closest center frequencies. The first and second crossover points between factors 1 and 2 are indicated as the first and second boundary frequencies; the crossover points between factors 2 and 3 are indicated as the third boundary frequencies. If the boundary frequencies are difficult to observe, they are indicated as unclear.

**Table 2:** **Boundary frequencies of the factor-related frequency bands observed in infants and adults**.

Language	Months of age	Boundaries (Hz)		
		First	Second	Third
Japanese	15	Unclear	Unclear	Unclear
	20	840	2900	5800
	24	840	2500	5800
English	15	Unclear	Unclear	Unclear
	20	Unclear	Unclear	Unclear
	24	840	2500	4800
Japanese	Adult	450	1850	3400
English	Adult	450	1600	2500

It appears that the same factors as in the infant speech shifted downward (leftward) in logarithmic frequency in the adult speech (Figure A3 in Appendix): The boundary frequencies (represented logarithmically) in the infant speech at 24 months were higher than those in the adult speech by a factor around 1.7 times. This indicates that the 24-month-old infants and the adult speakers used the articulation organs basically in the same way, and that the differences between the factor configurations were caused simply by the size differences – if the articulation organs are doubled in size, the frequencies indicating the factor locations are halved.

### Autocorrelation analyses

**Figure 2:**
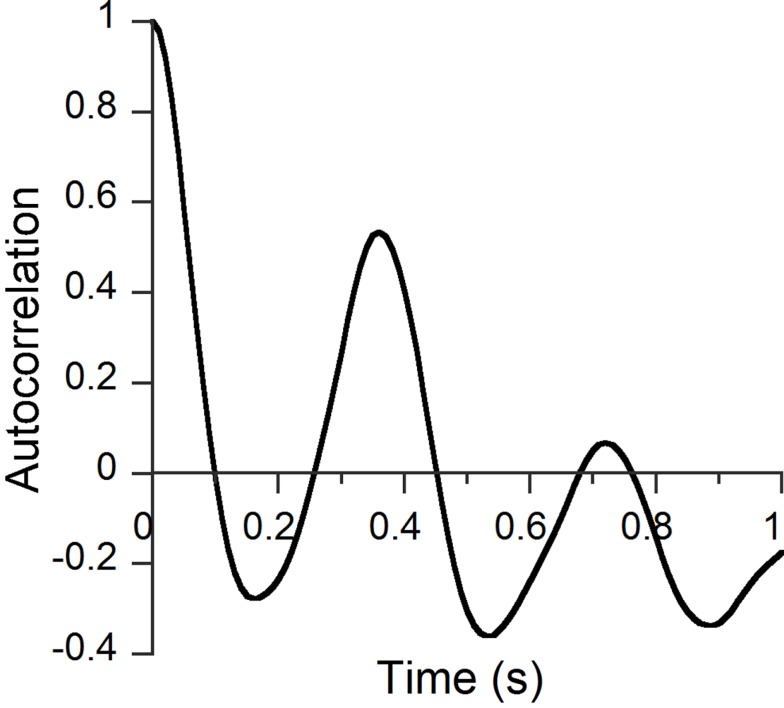
**An example of an autocorrelation graph for a Japanese-learning female infant at 24 months of age**.

We adopted the two-factor analysis, which produced visually clear results in most cases. The cumulative contributions were 23–27% for the two principal components. There was always a factor including a frequency range around 1600 Hz, which was similar to one of the factors in the three-factor analysis. Infants’ utterances ≥1.5 s were selected from speech samples so that at least 1500 factor scores, sampled at every 1 ms (as exactly as possible), were used for each autocorrelation analysis. Figure 2 shows an example of an autocorrelation function from a Japanese-learning female infant at 24 months of age. The amplitude of the first peak above zero (0.36 s in Figure 2) was taken as the representative autocorrelation score. If there was no peak above zero, the autocorrelation score was considered as without a peak. For Japanese-learning infants, the total numbers of utterances ≥1.5 s were 157, 141, and 311 at 15, 20, and 24 months of age, respectively. For English-learning infants, the total numbers of utterances ≥1.5 s were 118, 126, and 251 at 15, 20, and 24 months of age, respectively.

**Figure 3:**
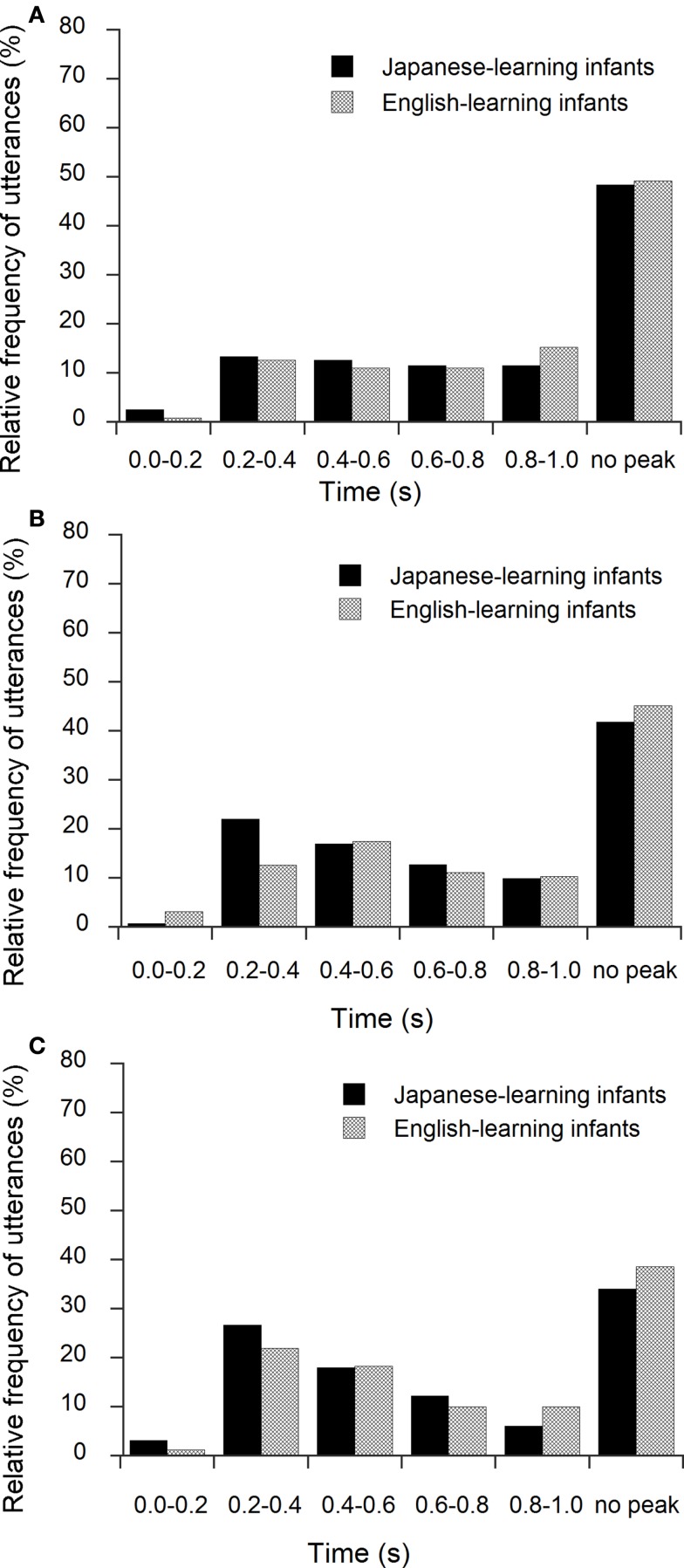
**Relative frequency distributions of the first peaks of autocorrelation in time for Japanese- and English-learning infants at 15 (A), 20 (B), and 24 (C) months of age**. The range 0.2–0.4 s, for example, does not include 0.2, but includes 0.4.

**Figure 4:**
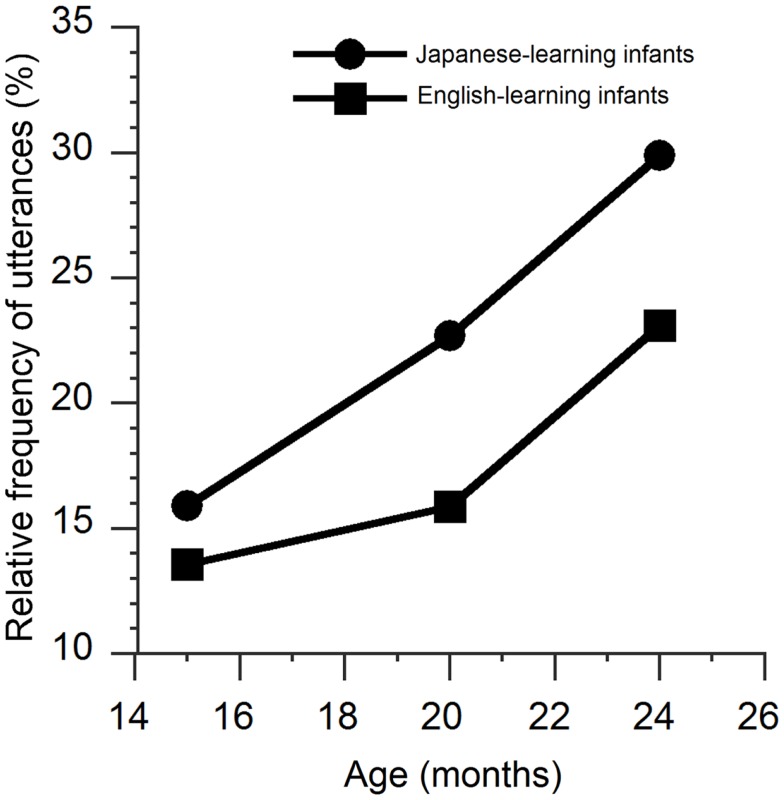
**Relative frequencies of the first peaks of autocorrelation located above 0.10 up to 0.40 s for Japanese- and English-learning infants across age (15, 20, and 24 months of age)**.

Figures 3A–C show the relative frequency distributions (%) of the first peaks for the Japanese- and English-learning infants at 15, 20, and 24 months of age. We focused on the first peaks located above 0.10 up to 0.40 s to explore the temporal periodicity, which was observed in previous studies (see, e.g., Kouno, 2001; Nathani et al., 2003; Dolata et al., 2008). As shown in Figure 4, 15.9, 22.7, and 29.9% of the first peaks were located in this range at 15, 20, and 24 months of age for the Japanese-learning infants, compared with 13.6, 15.9, and 23.1% for the English-learning infants. A chi-square test was carried out. For the Japanese-learning infants, the results showed that the relative frequency of the first peaks located above 0.10 up to 0.40 s increased across age, and the change was statistically significant (15, 20, and 24 months of age; χ^2^ = 11.35, *df* = 2, *p* < 0.01). There was a similar trend in the English-learning infants, but it was not statistically significant.

## Discussion

The purpose of the present investigation was to explore how the spectral fluctuations and the temporal periodicity of infant speech changed in Japanese- and English-learning infants between 15 and 24 months of age. The factor analyses of spectral fluctuations showed that three factors observed in adult speech appeared by 24 months of age in both linguistic environments. Those three factors were shifted to a higher range corresponding to the smaller vocal tract size of the infants (e.g., Goldstein, 1980; Vorperian et al., 2005). It is probable that the vocal tract structures of the infants had developed to adult-like configuration, but the whole vocal tract was still shorter than that of an adult. This corresponds to the vocal development study by Vorperian et al. (2005), which showed that the sizes of the various vocal tract structures grew rapidly to achieve 55–80% of that of the adult’s by 18 months of age. The results also agree with previous studies (e.g., Bond et al., 1982; Ishizuka et al., 2007), which showed there were rapid vowel space expansions during the first 2 years of age.

Autocorrelations were calculated from temporal fluctuations of the factor scores. It should be pointed out that the present study included a variety of utterances; it differs from previous studies, in which speech samples were limited to disyllabic vocalizations (e.g., Hallé et al., 1991; Vihman et al., 1998; Davis et al., 2000). One of the reasons that the previous analyses were limited to disyllabic vocalizations was the difficulty of measuring temporal periodicity. Conventional methods for adult speech, which are based on phonological properties, such as syllable structure and vowel reductions (e.g., Ramus et al., 1999; Low et al., 2000; Deterding, 2001; Grabe and Low, 2002; White and Mattys, 2007), were not applicable to infants. Since phonological properties in infant utterances are obscure. Thus, measuring duration was a common way to explore temporal periodicity in infant utterances. As Roach (1982) pointed out, there was no automatic measurement to identify stressed syllables: Phoneticians needed to judge stressed syllables by looking at speech waveforms, which might be influenced by incidental characteristics such as vowel length or pitch. The present authors employed an automatic method to identify temporal periodicity; it is based on temporal fluctuations of factor scores (by calculating autocorrelations). This method made it possible to explore the whole patterns of temporal periodicity in infant utterances. The amount of utterances with periodic nature of shorter time (up to 0.4 s) increased with age. The result corresponds to syllable durations observed in previous studies (e.g., Kouno, 2001; Nathani et al., 2003; Dolata et al., 2008). It needs to be examined whether this trend reflects ambient language rhythm.

In conclusion, the present analysis of spectral fluctuation showed that three factors observed in adult speech appeared by 24 months of age in both linguistic environments. Those three factors were shifted to a higher frequency range corresponding to the smaller vocal tract size. The amount of utterances with periodic nature of shorter time increased with age in both linguistic environments. This trend seemed clearer in the Japanese environment, which should be examined further in the future.

## Conflict of Interest Statement

The authors declare that the research was conducted in the absence of any commercial or financial relationships that could be construed as a potential conflict of interest.
